# Supplementary material for: Gastric epithelium from BRCA1 and BRCA2 carriers harbors increased double-stranded DNA damage and augmented growth
Source: bioRxiv. 2025 Oct 3:2025.10.01.679809. Preprint. [Version 1] doi: 10.1101/2025.10.01.679809 (PMC12621815; doi:10.1101/2025.10.01.679809)
Supplement: 1 [file NIHPP2025.10.01.679809V1-supplement-1.pdf]

bioRxiv preprint doi: https://doi.org/10.1101/2025.10.01.679809; this version posted October 3, 2025. The copyright holder for this preprint (which was not certified by peer review) is the author/funder, who has granted bioRxiv a license to display the preprint in perpetuity. It is made available under aCC-BY 4.0 International license.

Table S1. Baseline characteristics of the study cohort

Characteristic	Study Cohort	Control	*BRCA1*	*BRCA2*
n	15	5	5	5
Age (years), mean (SD)	63 (9.8)	59 (9.3)	68 (11.3)	60.8 (8.2)
Female sex, n (%)	7 (47%)	3 (60%)	2 (40%)	3 (60%)
White race, n (%)	15 (100%)	5 (100%)	5 (100%)	5 (100%)
Personal history of GC, n (%)	0 (0%)	0 (0%)	0 (0%)	0 (0%)
Personal history of other cancer, n (%)	7 (47%)	1 (20%)	2 (40%)	4 (80%)
Family history of GC, n (%)	1 (6.7%)	0 (0%)	1 (20%)	0 (0%)
Current or past *H. pylori* infection, n (%)	0 (0%)	0 (0%)	0 (0%)	0 (0%)

Table S2: Individual PDGO DNA sequencing data

		Germline alteration					*Allele specific copy number				
**PDGO ID**	**Gene**	**c.**	**p.**	**Pathogenicity**	**VAF score blood (%)**	**VAF score PDGO (%)**	**Sequenza copy number**	**CNVKit copy number**	**CIN (%)**	**MB (Mut/Mb)**	**HRD score**
GC087D_S32	*BRCA1*	4243delG	Glu1415Lysfs*4	Pathogenic	46.28	47.28	1,1	1,1	5.32	0.75	14
GC087P_S41	*BRCA1*	4243delG	Glu1415Lysfs*4	Pathogenic	46.28	50.06	1,1	1,1	2.54	0.75	21
GC089D_S40	*BRCA1*	4035delA	Glu1346Lysfs*20	Pathogenic	47.47	46.44	2,1	1,1	9.01	1.13	37
GC089P_S35	*BRCA1*	4035delA	Glu1346Lysfs*20	Pathogenic	47.47	48.16	2,1	1,1	1.31	1.13	33
GC090D_S25	*BRCA1*	5266dupC	Gln1756Profs*74	Pathogenic	50.74	45.01	1,1	1,1	5.59	1.88	13
GC090P_S28	*BRCA1*	5266dupC	Gln1756Profs*74	Pathogenic	50.74	47.35	1,1	1,1	22.24	1.51	11
GC092D_S24	*BRCA1*	5096G>A	Arg1699Gln	Pathogenic	44.81	51.52	1,1	1,1	2.65	2.26	18
GC092P_S19	*BRCA1*	5096G>A	Arg1699Gln	Pathogenic	44.81	46.45	1,1	1,1	0.10	0.38	21
GC093D_S4	*BRCA1*	4676-1G>A	Splice site	Pathogenic	46.84	47.30	1,1	1,1	23.08	1.51	28
GC093P_S36	*BRCA1*	4676-1G>A	Splice site	Pathogenic	46.84	49.48	1,1	1,1	24.95	0.38	30
GC043D_S27	*BRCA2*	4876_4877del	Asn1626Serfs*12	Pathogenic	47.08	45.35	1,1	1,1	26.11	2.64	21
GC043P_S31	*BRCA2*	4876_4877del	Asn1626Serfs*12	Pathogenic	47.08	43.29	1,1	1,1	25.87	2.26	15
GC067D_S6	*BRCA2*	EX13_23 copy gain	Copy gain	Pathogenic	n/a	n/a	1,1	1,1	1.55	1.13	22
GC067P_S20	*BRCA2*	EX13_23 copy gain	Copy gain	Pathogenic	n/a	n/a	1,1	1,1	6.35	3.77	23
GC073D_S30	*BRCA2*	5946delT	Ser1982Argfs*22	Pathogenic	42.97	47.90	1,1	1,1	5.07	1.13	15
GC073P_S15	*BRCA2*	5946delT	Ser1982Argfs*22	Pathogenic	42.97	46.11	1,1	1,1	12.17	0.38	16
GC080D_S37	*BRCA2*	5946delT	Ser1982Argfs*22	Pathogenic	45.33	46.74	1,1	1,1	33.68	1.13	30
GC080P_S42	*BRCA2*	5946delT	Ser1982Argfs*22	Pathogenic	45.33	45.19	1,1	1,1	32.81	1.51	36
GC081D_S38	*BRCA2*	5946delT	Ser1982Argfs*22	Pathogenic	43.68	46.20	2,2	1,1	2.39	5.28	27
GC081P_S33	*BRCA2*	5946delT	Ser1982Argfs*22	Pathogenic	43.68	44.26	2,2	1,1	1.74	3.39	43
GC070D_S23	Control	n/a	n/a	n/a	n/a	n/a	1,1	1,1	11.11	1.51	15
GC070P_S39	Control	n/a	n/a	n/a	n/a	n/a	2,1	1,1	8.07	1.51	29
GC085D_S22	Control	n/a	n/a	n/a	n/a	n/a	1,1	1,1	31.28	1.88	18
GC085P_S29	Control	n/a	n/a	n/a	n/a	n/a	1,1	1,1	13.08	1.51	20
GC094D_S26	Control	n/a	n/a	n/a	n/a	n/a	1,1	1,1	1.80	1.88	12
GC094P_S12	Control	n/a	n/a	n/a	n/a	n/a	2,0	1,1	5.13	1.13	20
GC095D_S21	Control	n/a	n/a	n/a	n/a	n/a	3,0	1,1	11.73	2.64	23
GC095P_S8	Control	n/a	n/a	n/a	n/a	n/a	1,1	1,1	0	1.51	17

*Allele specific copy number was generated by Sequenza and CNVKit. Data shown are number of a alleles, b alleles at the *BRCA1* or *BRCA2* locus.
